# Four-dimensional phase-contrast MRI with accelerated dual velocity encoding in patients with complex congenital heart disease: a feasibility study

**DOI:** 10.1093/ehjimp/qyag077

**Published:** 2026-04-28

**Authors:** Laurette Kalifa, Hichem Sakhi, Ana Beatriz Solana, Charles Roux, Guillaume Reverdito, Elodie Gouverneur, Daniel Sidi, Audrey Fels, Gilles Chatelier, Arshid Azarine

**Affiliations:** Department of Radiology, Hôpitaux Paris Saint-Joseph & Marie Lannelongue, 185 Rue Raymond Losserand, 75014, Paris, France; Department of Radiology, Hôpitaux Paris Saint-Joseph & Marie Lannelongue, 185 Rue Raymond Losserand, 75014, Paris, France; GE HealthCare, ASL Europe, Munich, Germany; Department of Interventional Radiology, AP-HP Sorbonne Université, Hôpital Universitaire Pitié-Salpêtrière, Paris, France; Department of Radiology, Hôpitaux Paris Saint-Joseph & Marie Lannelongue, 185 Rue Raymond Losserand, 75014, Paris, France; Department of Radiology, Hôpitaux Paris Saint-Joseph & Marie Lannelongue, 185 Rue Raymond Losserand, 75014, Paris, France; Department of Radiology, Hôpitaux Paris Saint-Joseph & Marie Lannelongue, 185 Rue Raymond Losserand, 75014, Paris, France; Department of Research and Medical Innovation, Hopitaux Paris Saint-Joseph & Marie Lannelongue, Paris, France; Department of Research and Medical Innovation, Hopitaux Paris Saint-Joseph & Marie Lannelongue, Paris, France; Department of Radiology, Hôpitaux Paris Saint-Joseph & Marie Lannelongue, 185 Rue Raymond Losserand, 75014, Paris, France

**Keywords:** 4D flow MRI, dual-VENC, congenital heart disease, Fontan

## Abstract

**Aims:**

To evaluate, in complex congenital heart disease (CHD), the feasibility of a single free-breathing dual-VENC 4D-flow MRI acquiring low- and high-VENC data consecutively within the same acquisition, and to quantify how metrics from low vs. high VENC co-vary across vascular territories.

**Methods and results:**

We conducted a single-centre, retrospective cohort study on a 3T system. Seventeen dual-VENC scans were acquired; 16 scans were analysable after quality review. We quantified the velocity-to-noise ratio (VNR), forward flow, peak velocity (Vmax), and the coefficient of variation (CV) of net flow across 30 cardiac frames in the ascending aorta (AAo), superior vena cava (SVC), inferior vena cava (IVC), pulmonary veins (PV), portal trunk (PT), and hepatic veins (HV). VNR was compared between VENC settings using a paired Wilcoxon signed-rank test. Agreement of forward flow and Vmax between low- and high-VENC was summarized with Spearman's rank correlation coefficient (*r*). Differences in variability (CV) were assessed with two-sided *F*-tests. Low-VENC increased VNR in AAo, PV, IVC, SVC, and PT (all *P* ≤ 0.003) and was not significant in HV (*P* = 0.54). CV was lower at low-VENC in AAo, PV, IVC, and SVC (all *P* ≤ 0.031). Agreement between low- and high-VENC was good-to-excellent in venous beds (SVC/IVC/PT *r* ≈ 0.67–0.88, all *P* ≤ 0.004), moderate in PV (*r* ≈ 0.57–0.62, *P* ≤ 0.024), but non-significant in AAo (*r* ≤ 0.55, *P* ≥ 0.17).

**Conclusion:**

Dual-VENC 4D-flow MRI is feasible and clinically useful in complex CHD. Low-VENC improves VNR and reduces variability for slow venous flows, while high-VENC is required to faithfully capture arterial jets. Together in a single acquisition, they extend velocity dynamic range and streamline comprehensive haemodynamic assessment.

## Introduction

Comprehensive haemodynamic assessment is central to decision-making in congenital heart disease (CHD).^1^ Conventional two-dimensional (2D) phase-contrast MRI provides robust through-plane flow quantification but is constrained by single-plane placement and prespecified velocity encoding (VENC), and it samples only a fraction of the circulation per examination.^2^ Four-dimensional (4D) flow MRI alleviates plane-selection bias by retrospectively interrogating any vessel within the acquired volume and has shown clinical utility in CHD, enabling volumetric flow mapping, valve assessment and visualization of complex patterns such as helices and vortices.^3–5^.

However, the single-VENC paradigm remains suboptimal when high-velocity arterial jets and slow venous flows must be interrogated within the same volume and cardiac cycle.^3^ If VENC is set too low, phase wraps produce aliasing in high-velocity regions; if too high, the phase accrual per unit velocity shrinks and the velocity-to-noise ratio (VNR) degrades, particularly in low-signal territories.^6^

To broaden measurable dynamic range, dual- or multi-VENC strategies combine a low-VENC acquisition (to enhance VNR and small-flow detectability) with a high-VENC acquisition (to avoid aliasing and preserve peak-velocity fidelity). Implementations range from sequential acquisitions fused in post-processing to integrated, accelerated sequences that interleave encodings within the same readout, as well as balanced multi-point schemes and triple-VENC approaches^7–13^. Their application has largely focused on arterial territories or relied on sequential scans or voxel-wise fusion strategies.

In contrast, the present study specifically addresses the clinical requirements of complex CHD by extending dual-VENC 4D-flow MRI to thoraco-abdominal circulations, including Fontan-related venous beds such as the hepatic veins (HV) and portal trunk. Moreover, our approach relies on a single free-breathing acquisition with back-to-back velocity encodings and preserves two independently reconstructed datasets rather than generating a fused velocity field, allowing a direct, territory-specific comparison of low- and high-VENC performance. Different vascular territories were intentionally selected to also reflect the diversity of circulatory systems involved in CHD, including systemic, pulmonary, and hepatic circulations. Finally, practical adoption in CHD requires solutions that minimize scan-time overhead and limit motion sensitivity.

Therefore, this present work evaluates an accelerated dual-VENC 4D-flow prototype that acquires low- and high-VENC encodings back-to-back within a single, free-breathing scan and reconstructs two independent datasets for side-by-side analysis. We hypothesized that, in patients with complex CHD, low-VENC would increase VNR and reduce frame-wise flow variability in venous territories, while high-VENC would remain necessary for arterial jets; taken together, these complementary roles would support the clinical practicality of dual-VENC as an one-stop strategy for comprehensive haemodynamic assessment.

## Methods

### Study design and population

This was a retrospective, single-centre cohort study of consecutive clinical cardiac magnetic resonance imaging examinations that included a dual-VENC 4D flow prototype between 2 February 2019 and 31 May 2021. Inclusion criteria comprised age ≥10 years, referral for MRI with flow assessment, and availability of dual-VENC 4D flow data. Non-inclusion criteria were legal incapacity or objection to data use; MRI or gadolinium contraindications. We excluded non-diagnostic examination due to poor image quality. All patients provided written informed consent to test the prototype sequence. The protocol was approved by the local ethics committee (IRB #00012157).

### MRI acquisition

**Figure 1:**
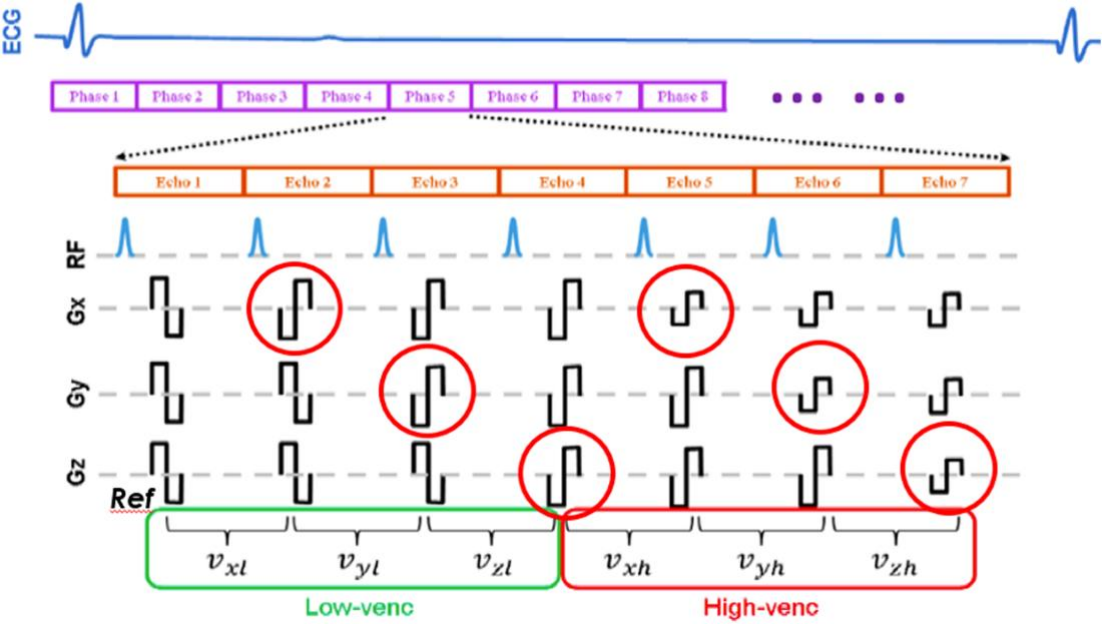
Dual-VENC PC-MRI acquisition and reconstruction. Each k-space line comprises seven TRs: one shared reference (Ref), three low-VENC flow-encoding directions (*x, y, z*), and three high-VENC directions (*x, y, z*). This single-scan scheme achieves 7 TR/line vs. 8 TR/line for two separate low- and high-VENC acquisitions.

All scans were performed on a 3T system (Discovery MR750, GE HealthCare, Chicago, IL, USA). The dual-VENC 4D flow prototype implemented back-to-back encoding blocks in 7 TRs per k-space line (shared reference + *x*/*y*/*z* low-VENC + *x*/*y*/*z* high-VENC), accelerated with kAt ARC (*Figure 1*). Typical parameters are summarized in *Table 1*: spatial resolution 2×2 × 2.2 mm^3^, temporal resolution 40–45 ms, 30 reconstructed cardiac phases, TR/TE ≈ 4.06/2.1 ms, flip angle 15°, field of view (FOV) 320–400 mm, and acceleration factor ≈ 7. VENC pairs were selected by the operator based on anticipated peak velocities (most commonly 100/300 cm s⁻1; full range 50/150 to 166/500 cm s⁻1). Retrospective ECG gating covered the full R–R interval. Respiratory motion control used a pencil-beam navigator at the liver–diaphragm interface. Contrast administration followed a triphasic 0.15 mmol kg⁻1 gadobutrol protocol (bolus, slow infusion 0.2 mL kg⁻1, saline flush), per institutional practice.

**Table 1:** Dual-VENC 4D flow MRI acquisition parameters

Parameter	Value
Scanner	3T (GE MR750)
Trajectory	Cartesian
Acceleration	k-t ARC (mean factor ≈7)
Spatial resolution	2×2 × 2.2 mm^3^
Temporal resolution	40–45 ms
Cardiac phases	30
TR/TE	≈4.06/2.1 ms
Flip angle	15°
Field of view (FOV)	320–400 mm
VENC pairs (cm·s⁻^1^)	Common 100/300 (range 50/150 to 166/500)
Respiratory motion	Navigator
ECG gating	Retrospective
Contrast	Gadobutrol 0.15 mmol·kg⁻^1^ (triphasic protocol)

### Cloud-based reconstruction and corrections

**Figure 2:**
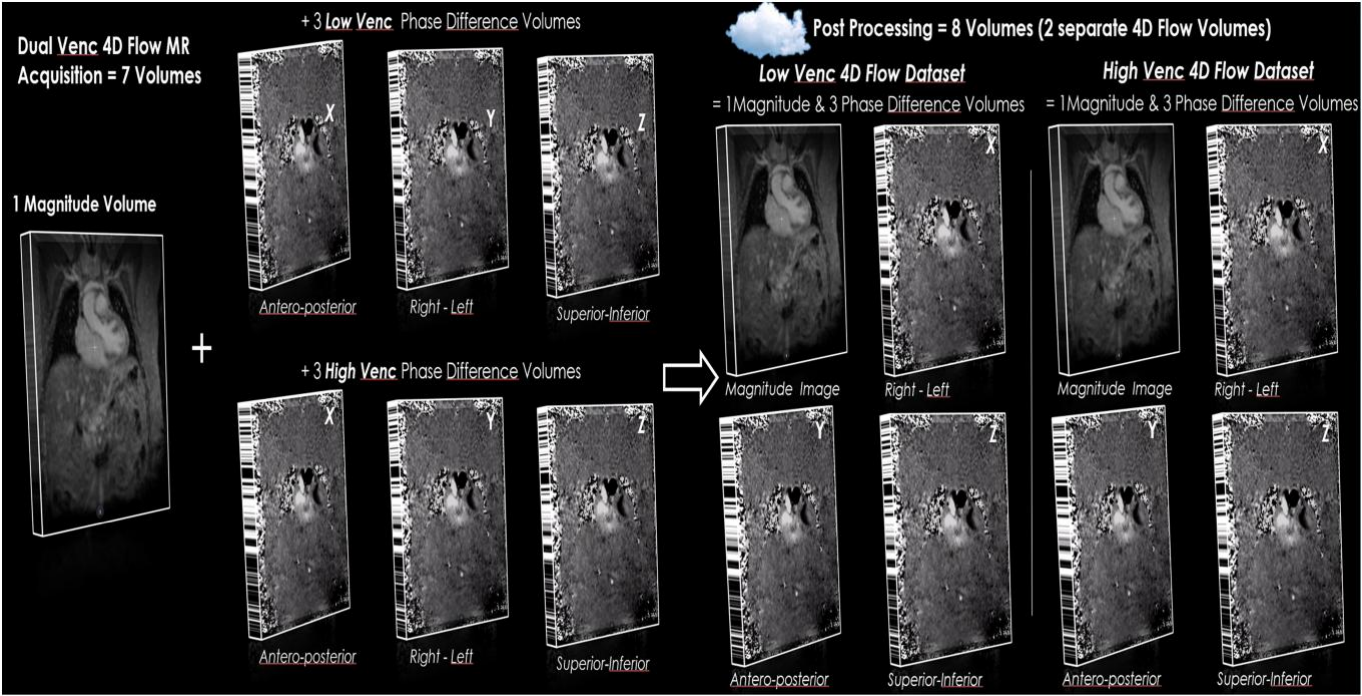
Dual-VENC 4D-flow image reconstruction and dataset generation. Seven raw volumes are acquired and uploaded to the cloud, where they are reconstructed into eight analysis volumes, yielding two fully independent datasets at low and high VENC. Background/eddy-current offset corrections and post-processing are performed separately for each dataset.

Magnitude (anatomical) and velocity images from each VENC were anonymized and uploaded to a cloud platform (Arterys, San Francisco, CA, USA) running on high-performance servers. The post-processing pipeline included volumetric eddy-current and background phase-offset correction using deep-learning-assisted identification of static tissue, producing two independent analysis datasets (low- and high-VENC, *Figure 2*). Users performed simultaneous multiplanar navigation and vessel-specific region of interest (ROI) placement in both datasets.

### Quantitative endpoints

**Figure 3:**
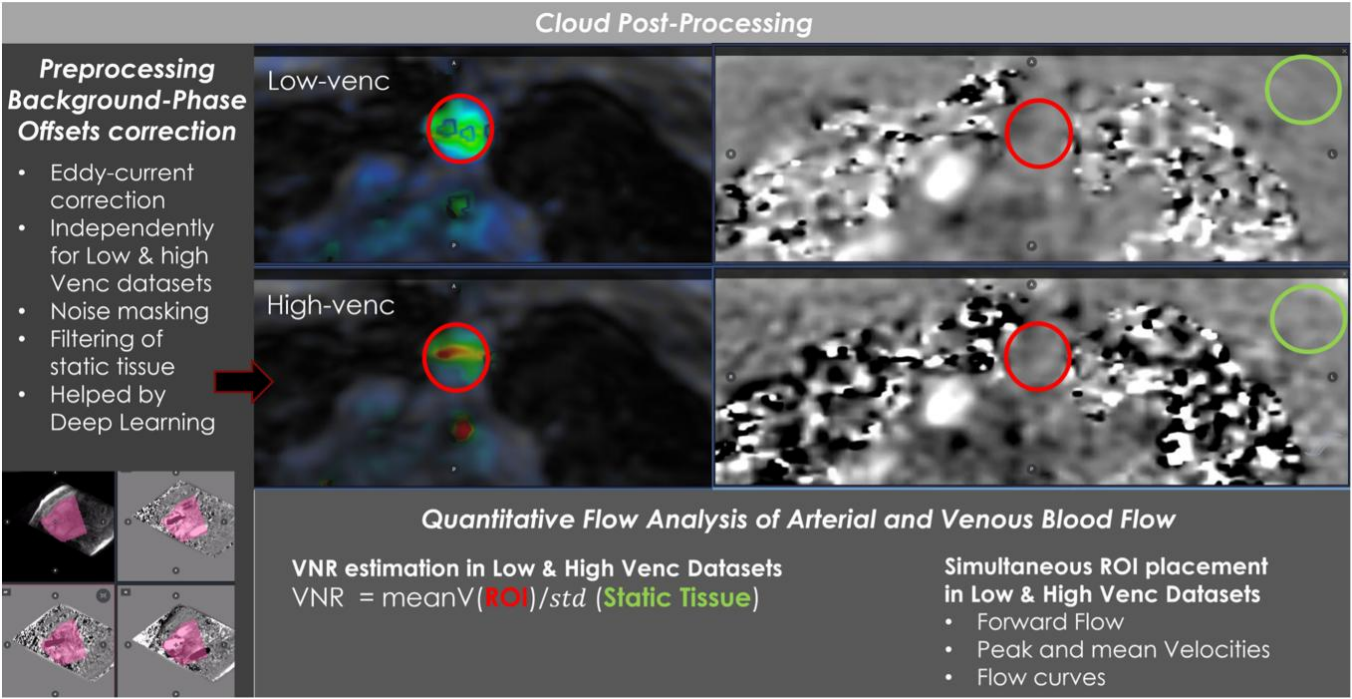
Cloud-based analysis workflow for low- and high-VENC datasets. Static-tissue masking (deep-learning-assisted) enables online background/offset correction. Vessel-specific ROIs are placed simultaneously on the low- and high-VENC volumes to quantify time-resolved flow and velocities. A static-tissue ROI (right panels) is used to measure phase noise (standard deviation) and compute the VNR.

For each vessel [ascending aorta (AAo), superior vena cava (SVC), inferior vena cava (IVC), pulmonary vein (PV), portal trunk (PT), HV)], matched circular ROIs were placed orthogonal to the vessel axis on reformat planes at standard anatomical levels (*Figure 3*). The following were extracted from both VENC datasets:

- VNR, estimated as mean peak velocity (Vmax) within the ROI divided by the standard deviation of velocity noise measured in a static-tissue ROI in the same volume.
- Forward flow (L min⁻^1^) over the cardiac cycle.
- Peak velocity (Vmax, cm s⁻^1^) over the cardiac cycle.
- Instantaneous net-flow variability, the coefficient of variation (CV) of the 30 time-resolved net-flow measures.

### Image quality

Two independent radiologists (blinded to VENC) graded diagnostic quality of magnitude images and angiograms on a 3-point scale (optimal, suboptimal, non-diagnostic) and recorded the presence of aliasing.

### Statistics

Superiority of VNR at low-VENC vs. high-VENC was tested with the paired Wilcoxon signed-rank test (two-sided, *α* = 0.05). Agreement between VENC settings for forward flow and Vmax was quantified with the Spearman rank correlation coefficient. Comparisons of frame-wise net-flow CV (low-VENC vs. high-VENC) were performed using *F*-tests of variance. Given the exploratory nature of this feasibility study and the small sample size, these analyses were intended to provide a pragmatic comparison of variability between low- and high-VENC conditions rather than a formal inference framework.

## Results

### Study population and feasibility

Seventeen patients underwent dual-VENC 4D flow MRI; one was excluded due to non-diagnostic image quality (final *n* = 16). The median age was 29 years (IQR 18–22), 78% male. CHD substrates included mainly single ventricle (*n* = 8), transposition of the great arteries with atrial switch (*n* = 2) or congenitally corrected transposition of the great arteries (*n* = 3), and other lesions such as VSD (*n* = 3) or aortic coarctation (*n* = 1). All dual-VENC scans were acquired successfully in 18 ± 3 min (median ∼15 ± 4 min), ∼1.5× the scan time of a standard single-VENC 4D flow, hence, shorter than two separate acquisitions.

### Velocity-to-noise ratio

Low-VENC systematically improved VNR across most vascular territories (*Table 2*). Statistically significant improvements were observed in the AAo (*P* = 0.039), PV (*P* = 0.003), IVC (*P* < 0.001), SVC (*P* = 0.003), and PT (*P* < 0.001), whereas no significant difference was found in HV (*P* = 0.54). Visually, low-VENC PC-MRA offered crisper depiction of venous anatomy according to both observers.

**Table 2:** Summary of quantitative metrics at low- and high-VENC by vessel

	Vmax Low-V	Vmax High-V	F-Flow Low-V	F-Flow High-V	VNR Low-V	VNR High-V	Net flow variation coefficient low-V	Net flow variation coefficient high-V
*AAo*								
Mean (SD)	66.23 (18.85)	81.00 (28.78)	49.19 (18.20)	49.02 (17.61)	2063.62 (617.15)	1490.12 (957.29)	1.42 (0.21)	1.64 (0.33)
Median [Q1; Q3]	65.94 [59.23; 75.04]	73.57 [64.61; 90.86]	51.48 [33.17; 60.69]	51.13 [43.01; 59.44]	1941.51 [1859.95; 2084.29]	1172.03 [1033.44; 1547.65]	1.37 [1.32; 1.56]	1.75 [1.42; 1.80]
*r* correlation coefficient (*P*)	0.29 (0.50)		0.55 (0.17)					
*P* *					**0.039**		**0.031**	
*PV*								
Mean (SD)	44.65 (11.64)	47.69 (17.21)	13.97 (8.53)	11.12 (8.04)	1650.72 (734.52)	951.05 (824.34)	0.65 (0.22)	1.54 (2.73)
Median [Q1; Q3]	44.28 [36.75; 46.97]	44.53 [34.91; 53.66]	12.21 [8.50; 16.03]	9.25 [6.17; 13.73]	1606.12 [1088.44; 2226.73]	577.16 [409.04; 1657.86]	0.60 [0.50; 0.70]	0.84 [0.70; 1.16]
*r* correlation coefficient (*P*)	**0.57 (0.024)**		**0.62 (0.013)**					
*P* *					**0.003**		**0.002**	
*IVC*								
Mean (SD)	43.08 (19.03)	42.83 (19.23)	23.19 (14.17)	17.67 (9.66)	1453.43 (936.48)	863.54 (450.62)	0.78 (0.83)	1.59 (3.21)
Median [Q1; Q3]	40.18 [26.94; 56.01]	41.75 [28.64; 52.31]	19.45 [14.29; 30.82]	15.36 [10.18; 23.66]	1154.25 [959.05; 1763.44]	804.00 [642.28; 969.14]	0.53 [0.19; 1.10]	0.56 [0.36; 1.22]
*r* correlation coefficient *(P)*	**0.76 (0.001)**		**0.86 (<0.001)**					
*P* *					**<0.001**		**0.021**	
*SVC*								
Mean (SD)	41.91 (21.06)	48.80 (22.69)	21.60 (13.62)	24.35 (15.45)	1623.42 (973.23)	1024.05 (768.99)	0.49 (0.26)	0.41 (0.20)
Median [Q1; Q3]	40.58 [23.88; 43.35]	47.00 [26.84; 59.19]	17.38 [13.36; 25.25]	21.16 [13.50; 27.12]	1263.90 [885.57; 2253.35]	724.52 [488.57;1378.72]	0.47 [0.27;0.64]	0.44 [0.30;0.52]
*r* correlation coefficient *(P)*	**0.88 (<0.001)**		**0.84 (<0.001)**					
*P* *					**0.003**		**0.003**	
*PT*								
Mean (SD)	14.74 (4.31)	16.11 (4.32)	5.71 (3.04)	5.50 (3.27)	683.86 (511.76)	423.81 (356.55)	0.18 (0.24)	0.23 (0.28)
Median [Q1; Q3]	15.32 [12.29; 17.57]	16.40 [14.64; 17.33]	5.24 [3.92; 7.60]	4.60 [3.76; 6.94]	627.14 [336.64; 836.18]	335.71 [196.58; 508.16]	0.09 [0.07; 0.15]	0.12 [0.07; 0.17]
*r* correlation coefficient (*P*)	**0.76 (0.001)**		**0.67 (0.004)**					
*P* *					**<0.001**		0.08	
*HV*								
Mean (SD)	14.65 (7.71)	17.18 (7.00)	2.02 (1.06)	2.93 (1.67)	408.77 (295.05)	293.48 (118.62)	0.89 (1.40)	1.28 (1.47)
Median [Q1; Q3]	12.80 [8.77; 21.27]	14.40 [11.39; 24.02]	1.85 [1.25; 2.67]	2.46 [2.13; 3.22]	432.83 [195.75; 505.32]	340.06 [191.22; 399.39]	0.36 [0.16; 1.10]	0.42 [0.17; 1.91]
*r* correlation coefficient (*P*)	**0.80 (0.001)**		0.32 (0.26)					
*P* *					0.54		0.45	

AAo, ascending aorta; F-Flow, forward flow; High-V, high-Venc; HV, hepatic veins; IVC, inferior vena cava; Low-V, low-Venc; PT, portal trunk; PV, pulmonary veins; SVC, superior vena cava; VENC, velocity encoding; VNR, velocity-to-noise ratio; Vmax, peak velocity.

*R* is the Spearman correlation coefficient given with his *P*-value (*P* < 0.05 = difference is statistically significant)

^*^Reported *P*-values are from paired Wilcoxon tests comparing low vs. high VENC for VNR and *F*-variance tests for Net Flow variation coefficient (*P* < 0.05 = difference is statistically significant).

### Forward flow and Vmax

Agreement between low- and high-VENC measurements was strong for systemic and pulmonary veins, including the SVC (forward flow *r* = 0.88; Vmax *r* = 0.84, both *P* < 0.001), IVC (*r* = 0.76 and 0.86, *P* ≤ 0.001), and PT (*r* = 0.76 and 0.67, *P* ≤ 0.004). Agreement was moderate for PV (*r* = 0.57 and 0.62, *P* = 0.024 and 0.013, respectively). In contrast, concordance was weak and non-significant in the AAo (*r* = 0.29 and 0.55, *P* = 0.50 and 0.17).

### Instantaneous net-flow variability across the cardiac cycle

Low-VENC significantly reduced frame-wise net-flow variability, as assessed by the CV. *F*-tests demonstrated lower variability at low-VENC in the AAo (*P* = 0.031), PV (*P* = 0.002), IVC (*P* = 0.021), and SVC (*P* = 0.003). Differences were not significant in the PT (*P* = 0.08) or HV (*P* = 0.45).

### Image quality and artefacts

Two blinded observers graded overall image quality as optimal in 14/17, suboptimal in 2/17, and non-diagnostic in 1/17 (excluded). Image quality assessment showed good qualitative inter-observer agreement, with any discrepancies resolved by consensus. Velocity aliasing was more frequent in arterial measurements at low-VENC than at high-VENC (as expected) but did not preclude quantitative analysis when the paired high-VENC dataset was available for anti-aliasing or corroboration.

## Discussion

This feasibility study shows that low-VENC improves measurement quality—higher VNR and lower frame-wise flow variability—particularly in slow-flow venous territories, while high-VENC remains essential to faithfully capture arterial peak velocities prone to aliasing. In the final tables, Spearman's rank coefficient demonstrated good-to-excellent concordance across VENCs in veins (SVC, IVC, PT; moderate in PV) but weak, non-significant correlations in the AAo, underscoring the complementary value of high-VENC for arterial jets. The lack of correlation between low- and high-VENC in the AAo reflects the known limitations of low-VENC encoding for high-velocity arterial jets rather than a failure of the dual-VENC strategy. These findings reinforce the need for high-VENC data for reliable arterial peak velocity quantification and caution against the isolated use of low-VENC measurements in the AAo. Together, these results support the dual-VENC paradigm: a single examination that secures both regimes rather than choosing one at the expense of the other.

Simultaneous access to both regimes within a single examination is operationally attractive in complex CHD follow-up, where arterial stenoses or regurgitant jets coexist with slow venous flows critical to clinical decision-making (e.g. Fontan pathway, hepatic venous congestion, portal haemodynamics). Cloud-based processing and correction workflows handled the large raw datasets and enabled practical, side-by-side analysis of both VENC datasets.

### Comparison with previous studies and multi-VENC alternatives

Our findings align with prior multi-VENC work showing that combining encodings broadens measurable velocity range and improves data quality. Multi-VENC/dual-VENC implementations have reported good agreement with 2D phase-contrast flow and favourable scan–rescan/inter-observer reproducibility, alongside improved visualization of complex flow patterns and pathlines compared with single-VENC 4D-flow.^7^ Post-processing strategies that fuse separately acquired low- and high-VENC datasets can recover the low-VENC VNR while unwrapping aliasing, but they remain vulnerable to inter-scan motion and registration error.^8,13^ Hardware-/sequence-level solutions such as five-point balanced flow encoding and triple-VENC increase VNR and dynamic range with modest time penalties when combined with acceleration.^10,14^ Accelerated dual-VENC with a shared reference and kAt undersampling has also been demonstrated, achieving 7 TR per line and enabling neurovascular and cardiovascular applications.^9,12,15^

Within this landscape, the present study differs in several practical aspects relevant to CHD. First, we extend dual-VENC 4D-flow MRI to a thoraco-abdominal setting in patients with complex CHD, explicitly including Fontan-related venous circulations such as the HV and portal trunk, which have been minimally explored in prior work. Second, low- and high-VENC data are acquired consecutively within a single free-breathing scan, limiting motion sensitivity, and scan-time overhead compared with sequential acquisitions. Third, rather than generating a single fused velocity field, we intentionally preserved two independently reconstructed datasets, enabling side-by-side analysis of VNR, flow measurements, and variability according to vascular territory.

Consistent with prior reports, we observed that low-VENC enhances VNR and reduces frame-wise flow variability in slow-flow venous beds, whereas high-VENC is required for arterial jets in the AAo. These complementary roles support dual-VENC as a pragmatic alternative to sequential acquisitions or purely post-processed multi-VENC, mitigating motion sensitivity while keeping acquisition time close to a single 4D-flow.

### Clinical implications

In complex CHD follow-up, a single dual-VENC 4D-flow acquisition can:

1. deliver aliasing-safe velocity quantification for arterial jets (stenoses, regurgitant flows) via high-VENC, and
2. provide higher-precision venous assessment (SVC/IVC, HV, PT, PV) via low-VENC, where better VNR and lower variability improve confidence in small flow measurements differences but also for further secondary flow analysis and measurements such as recirculation, vorticity, and viscous energy dissipation, where noise level should be reduced (e.g. Fontan pathway).
3. Operationally, dual-VENC avoids scheduling two scans and enables side-by-side review of both datasets, streamlining interpretation, with the same amount of contrast media injection.

As 4D-flow MRI continues to gain indications both within CHD and across broader cardiovascular practice—with emerging applications in valvular disease, aortic pathology, cardiomyopathy, venous haemodynamics—ensuring a robust acquisition is crucial; when slow and fast flows coexist, dual-VENC provides the practical foundation for reproducible, decision-grade quantification.^16,17^ Therefore, to facilitate broader clinical adoption and research applications, manufacturers should develop more powerful 4D flow MRI post-processing tools specifically optimized for managing the large datasets provided by dual-VENC sequences.

### Limitations

This study is a single-centre feasibility investigation with a limited sample size and without a prospective single-VENC comparator or an external reference standard, which may limit the generalizability of the findings. Acquisition parameters were optimized for thoracic and hepatic vessels, potentially compromising spatial resolution; finer FOV adjustments could improve resolution and/or reduce scan time. VENC selection remains operator-dependent, requiring prior knowledge of peak velocities to avoid aliasing at low-VENC settings. Furthermore, no robust, purpose-built 4D flow software exists to efficiently post-process the large datasets generated. We adapted our cloud-based Pixel AI (Tempus) software for simultaneous analysis of both datasets, but there is a need for a fluent dedicated solution specifically the fusion of low-VENC and high-VENC datasets acquired simultaneously.

Furthermore, the comparison of coefficients of variation relied on *F*-tests of variance, which assume normality and independence of variance estimates. These assumptions may be imperfectly met given the modest sample size, repeated measures within subjects, and potentially non-Gaussian flow distributions. Accordingly, these results should be interpreted as exploratory. Nevertheless, the observed reduction in variability at low-VENC was directionally consistent across multiple vascular territories, supporting the robustness of the overall findings.

The CHD cohort was heterogeneous, encompassing a range of diagnoses and circulatory configurations. While this heterogeneity precludes disease-specific conclusions, it reflects real-world CHD practice and was considered appropriate for this feasibility study. Indeed, dual-VENC 4D-flow MRI may be particularly advantageous in heterogeneous settings, where markedly different flow regimes coexist within the same acquisition volume.

Finally, this study did not include an external reference standard such as 2D phase-contrast MRI or invasive catheter measurements. As such, it does not aim to establish absolute accuracy of flow or velocity quantification. Rather, it focuses on the internal consistency and clinical feasibility of a dual-VENC 4D-flow strategy in complex CHD. In this context, agreement and directional differences between low- and high-VENC datasets are used as surrogate indicators of data quality and robustness.

## Conclusions

Accelerated dual-VENC 4D flow MRI is feasible in complex CHD follow-up, providing improved VNR and reduced flow-dispersion for slow-flow venous territories while preserving measurement fidelity in high-velocity arterial jets.

## Consent

All participants (or legal guardians) provided written informed consent for the prototype sequence and research use of de-identified data. The study was approved by the institutional review board (IRB #00012157) and conducted in accordance with the Declaration of Helsinki.

## Data Availability

Raw and derived data underlying this article contain personal health information and cannot be shared publicly due to institutional restrictions. De-identified data supporting the findings are available from the corresponding author on reasonable request and with ethics approval.

## Lead author biography

Dr Laurette Kalifa is a radiologist specializing in cardiovascular imaging. She practices at Hôpital Marie-Lannelongue and Hôpital Paris Saint-Joseph, where she is actively involved in the diagnosis and imaging follow-up of patients with congenital heart diseases. Her expertise spans a wide range of advanced imaging techniques, including cardiac MRI and cardiac CT, with a particular interest in 4D Flow imaging for comprehensive assessment of cardiovascular haemodynamics.
